# Supplementary material for: KIRA6 is an Effective and Versatile Mast Cell Inhibitor of IgE‐mediated Activation
Source: Eur J Immunol. 2024 Dec 15;55(2):e202451348. doi: 10.1002/eji.202451348 (PMC11830387; doi:10.1002/eji.202451348)
Supplement: Supplementary file 1 — Supporting Information [file EJI-55-e202451348-s001.pdf]

Suppl. Fig. 1: Effect of KIRA8 on XBP1 splicing in HMC-1.2 cells.

HMC-1.2 cells were pre-incubated with the solvent control DMSO or the indicated KIRA8 concentrations for 1 h followed by 5 µg/mL Tunicamycin (TM) treatment for 3 h and the expression of *XBP1s* was measured by RT-qPCR. *HPRT* served as housekeeping gene (n=3).

The data presented are derived from 3 independent experiments, each comprising 3 technical replicates for all 6 samples.

Data are shown as mean +/- SD. Symbols indicate individual values of biological replicates. RM one-way ANOVA followed by Dunnett's test to correct for multiple comparison. * p<0.05, ** p<0.01, *** p<0.001, **** p<0.0001, ns indicates no significance.

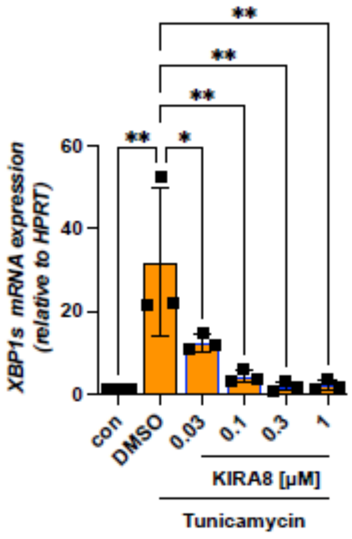

Suppl. Fig. 2: KIRA6 reduced Ca^{2+} mobilization of antigen-stimulated WT BMMCs.

WT BMMCs were pre-incubated with the indicated KIRA6 concentrations for 30 min and

Ca^{2+} mobilization was measured for 4 min by flow cytometry using Ca^{2+}-sensitive fluorescent dyes Fluo-3 and Fura Red. Steady-state fluorescence was measured for 1 min before 20 ng/mL DNP-HSA (arrow mark) were added.

Data are representative graphs of 3 independent experiments, each comprising 3 technical replicates for all indicated samples.

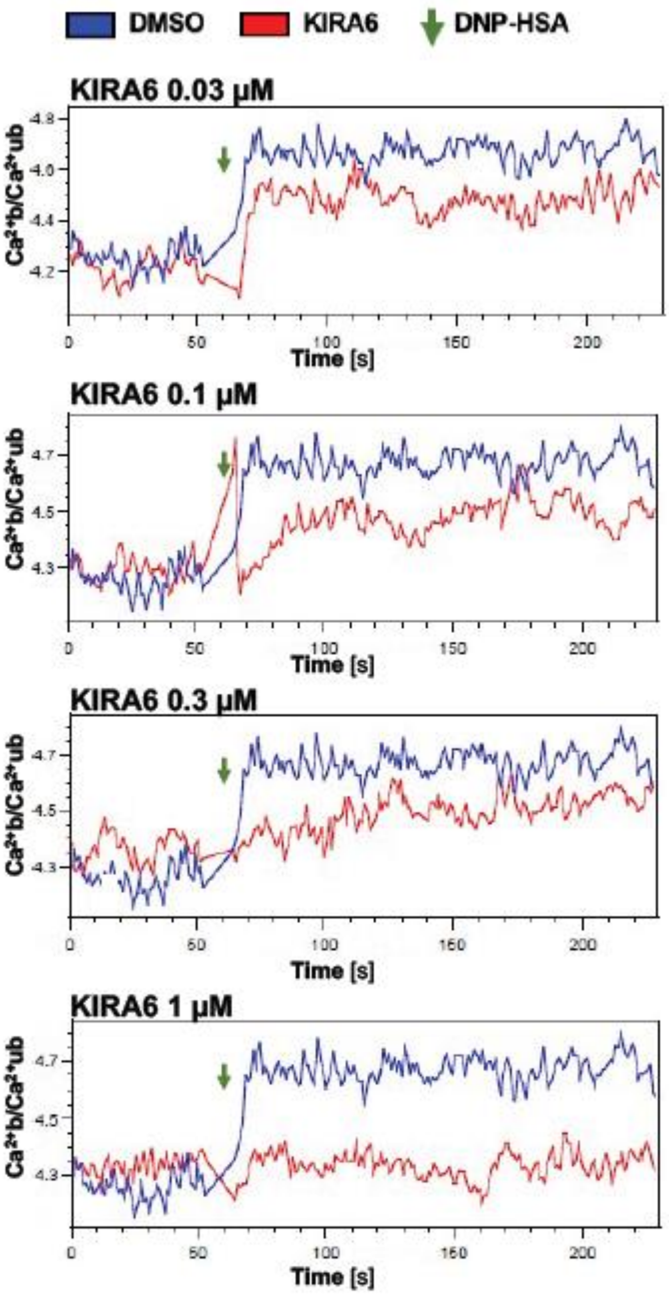

Suppl. Fig. 3: KIRA6 reduces FcεRI-dependent signaling in *Ship1*$^{-/-}$ BMMCs.

IgE pre-loaded *Ship1*$^{-/-}$ BMMC were incubated with the solvent control DMSO or 1 µM KIRA6 for 1 h prior to stimulation with 20 ng/mL DNP-HSA for indicated time. (**A**) Global tyrosine phosphorylation was detected after DNP-HSA stimulation at the indicated time points in DMSO- or KIRA6-pretreated BMMCs on WB using 4G10 antibodies. LYN was detected on the same WB membrane using an anti-LYN antibody (n=3).(**B**) DNP-HSA-induced phosphorylation of PKB, MEK1/2 and ERK1/2 in DMSO- or KIRA6-pretreated cells was detected in whole cell lysates by WB using phospho-specific antibodies. P85 served as a loading control. Numbers indicate mean values obtained from densitometry analyses (n=3). (**A**, **B**) The data show representative Western blots of 3 independent experiments for all indicated samples.

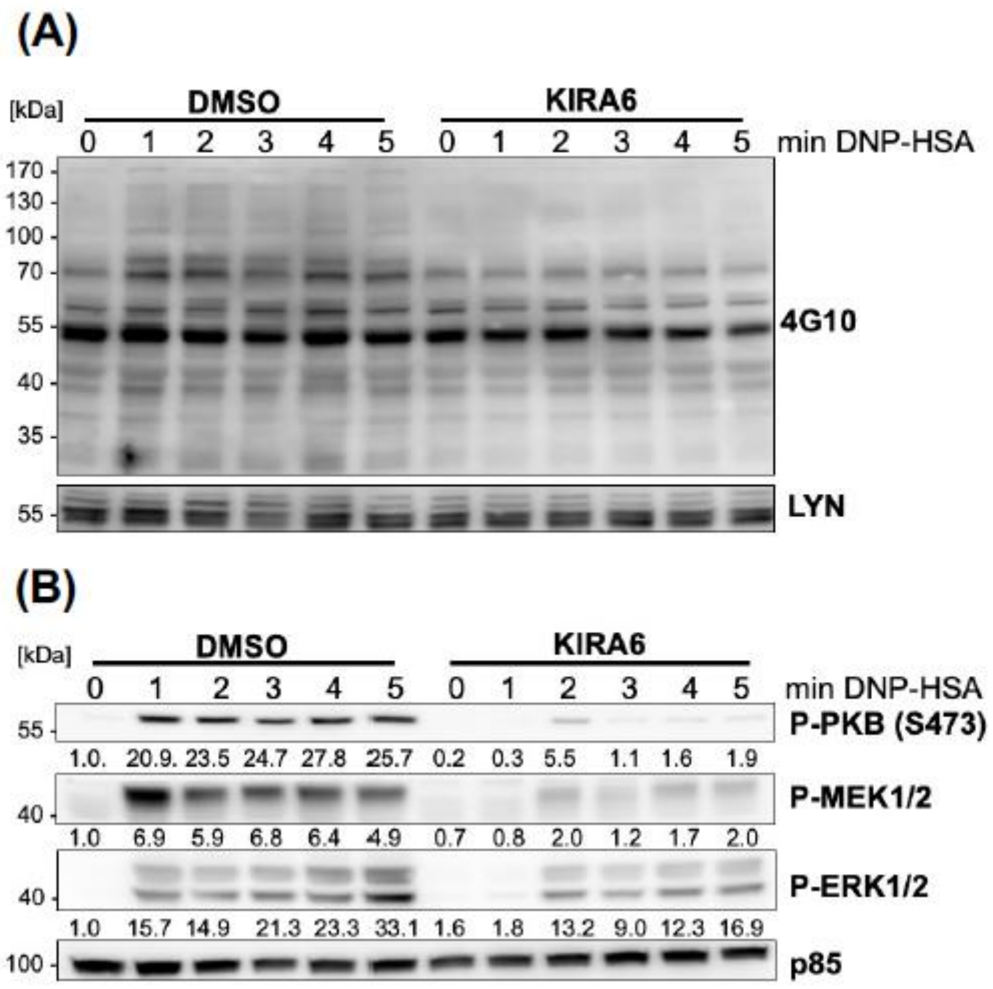

Suppl. Fig. 4: Evaluation of KIRA6 binding to LYN and LYN kinase activity in *in vitro* assays.

(**A**)Thermal shift assay with LYN in absence (green) and presence of 100 µM KIRA6 (red) indicating a destabilization of LYN (by 3.1°C) in presence of KIRA6 (n=2).

(**B**)Thermal shift assay with FYN in absence (blue) and presence of 100 µM KIRA6 (red) indicating a destabilization of FYN (by 3.0°C) in presence of KIRA6 (n=2).

(**C**) The IC50 calculator webtool (https://www.aatbio.com/tools/ic50-calculator) was used to calculate the IC50 of KIRA6 from the in vitro kinase assay data shown in Fig. 3 C and D (n=3).

(**A, B**) Thermal shift assay data were obtained from two independent experiments, each evaluating two distinct conditions. Data shown in Fig. 3 (**C**) and (**D**) are from 3 independent Western blot experiments with 7 samples that were used to calculate the IC50.

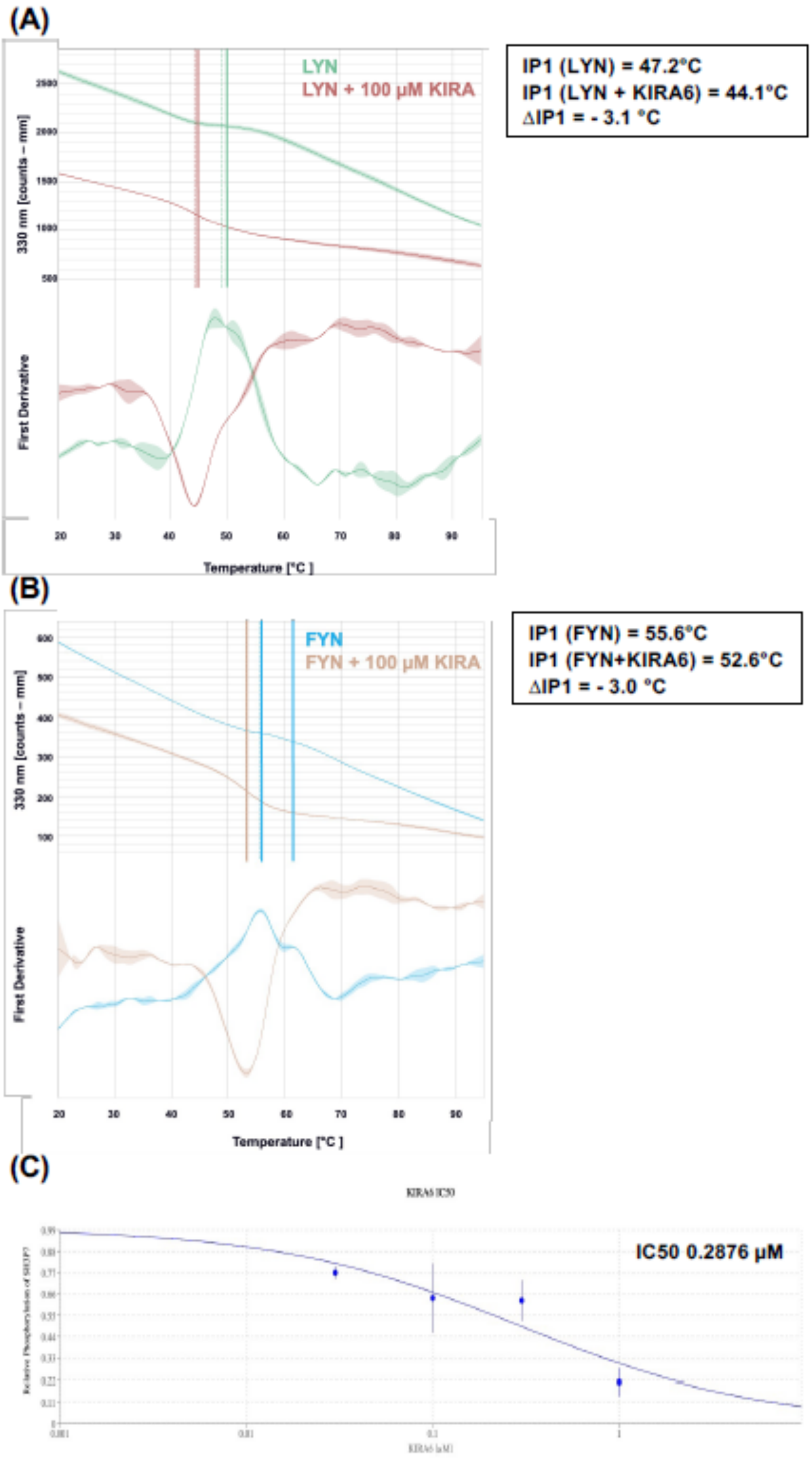

Suppl. Fig. 5: Impact of KIRA6 on FcεRI independent mast cell function in WT and Lyn$^{-/-}$ BMMCs.

WT BMMC (gray bars) or *Lyn*$^{-/-}$ BMMCs (white bars) were incubated with the solvent control DMSO or the indicated KIRA6 (red line) concentrations for 1 h prior to treatment with 100 nM thapsigargin (TG) for 4 h (all n=3). (**A**) *Xbp1s* and (**B**) *Il6* expression was detected by RT-qPCR in WT BMMCs. (**C**) *Xbp1s* or (**D**) *Il6* expression was detected by RT-qPCR in *Lyn*$^{-/-}$ BMMCs. (**E**) and (**F**) WT BMMC (gray bars) or *Lyn*$^{-/-}$ BMMCs (white bars) were incubated with the solvent control DMSO or the indicated KIRA6 (red line) concentrations for 1 h prior to treatment with 100 nM thapsigargin (TG) for 4 h and (**E**) Secreted IL-6 (n=3) and (**F**) TNF (n=3) was measured by ELISA assays.

(**A**, **B**, **C**, **D**, **E**, **F**) The data presented are derived from 3 independent experiments, each comprising 3 technical replicates for all indicated samples.

Data are shown as mean +/- SD. Symbols indicate individual values of biological replicates.

(A), (B), (C), (**D**) RM one-way ANOVA followed by Dunnett's test to correct for multiple comparison. (**E**), (**F**) Two-way ANOVA followed by Dunnett's multiple comparisons test.

* p<0.05, ** p<0.01, *** p<0.001, **** p<0.0001, ns indicates non significance.

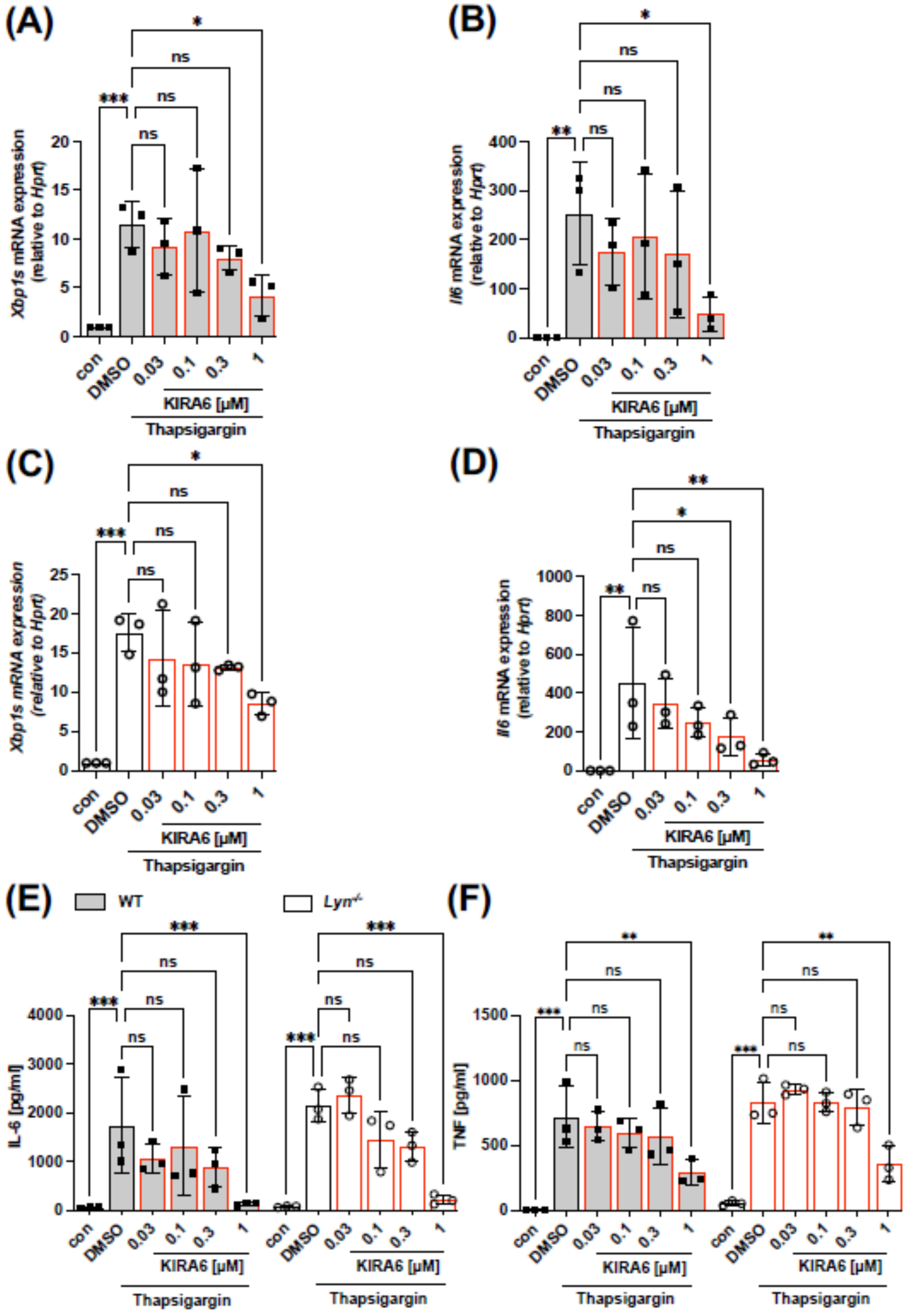

Suppl. Fig. 6: Impact of KIRA6 on FcεRI independent mast cell function in rat PCLS and the expression of SRC family kinases in WT BMMCs.

(**A**) Rat precision-cut lung slices were incubated with the solvent control DMSO or the indicated KIRA6 concentrations for 10 min following challenges with methacholine for 15 min and the airway area after bronchoconstriction was determined (n=12).

(**B**) Transcriptomic data from 3 independent analyses were used to depict mRNA expression of different SRC family kinases in WT BMMCs (n=3).

(**C**) Tryptase levels of peritoneal MCs were assed in a direct peritonitis model. KIRA6 (5 mg/kg and 10 mg/kg) was administered via intraperitoneal injection in mice 30 minutes prior to anti-mouse IgE injection. Mice were euthanized 30 minutes post-IgE injection and peritoneal lavages (2 mL) were collected. Tryptase activity was measured using an artificial, specific substrate (n=4).

(**A**) 12 Precision-cut lung slices (samples) were used for the three KIRA6 data points and 27 precision-cut lung slices (samples) for the control data point. (**B**) The data are from 3 independent experiments with 3 biological replicates in each experiment for all 5 samples.

(**C**) The data are from the peritoneal lavages from 4 mice (samples), each comprising three technical replicates for all 4 samples.

Data are shown as mean +/- SD. Symbols indicate individual values of biological replicates. (**A**) Ordinary one-way ANOVA followed by Dunnett's test to correct for multiple comparison. (**B**)Two-way ANOVA followed by Dunnett's multiple comparisons test. * $p<0.05$, ** $p<0.01$, *** $p<0.001$, **** $p<0.0001$, ns indicates no significance.

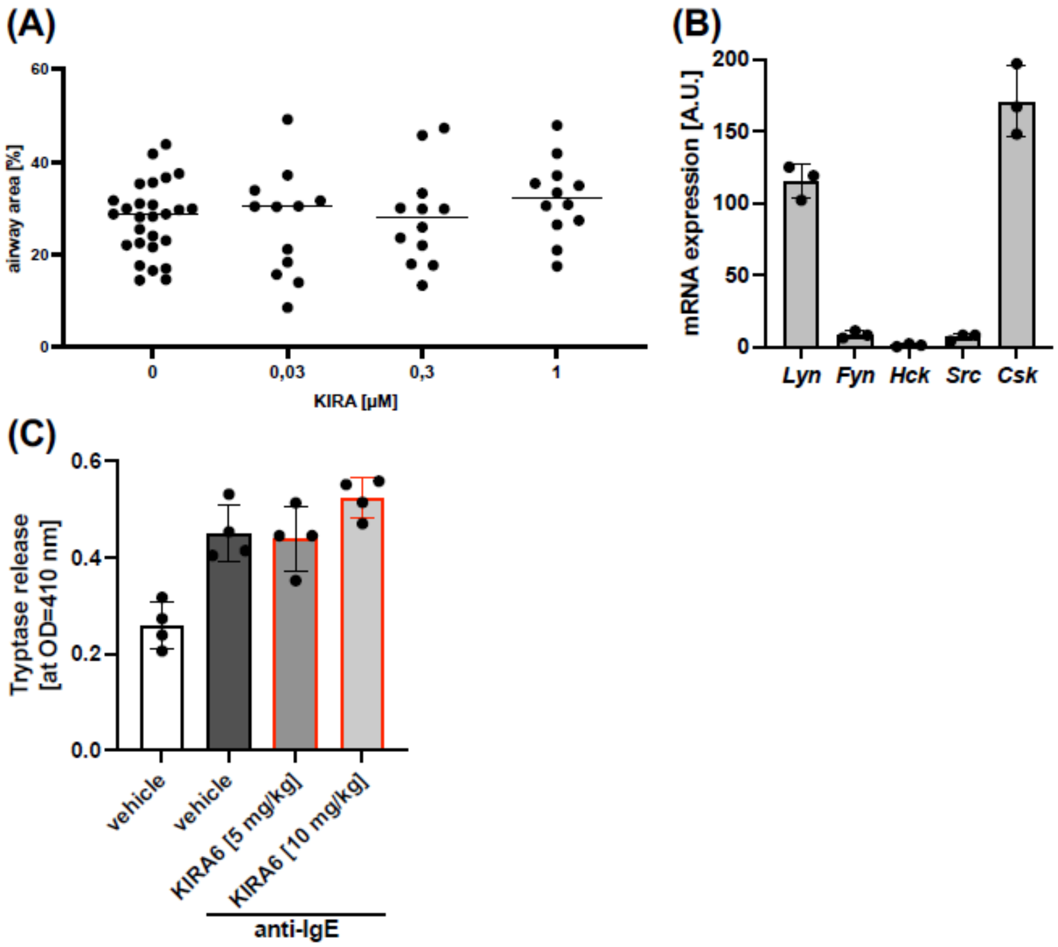

Suppl. Fig. 7: Evaluation of the cytotoxicity of KIRA6 on WT and *Ship1$^{-/-}$* BMMCs

The impact of KIRA6 on proliferation, metabolic activity and viability was evaluated in WT (gray bars) and *Ship1$^{-/-}$* (white bars) BMMCs. Cells were seeded at a density of 5 x 10^5 cells/mL and treated with the solvent DMSO or the indicated KIRA6 concentrations for 24 h (A, B) or 72 h (C-F).

(**A, B**) Cell viability and (**C**, **D**) proliferation was measured by automated multi-parameter cell counting using a Casy cell counter (Innovatis) (all n=3). (**E**, **F**) Metabolic activity was measured using the XTT Cell Proliferation Kit II (Roche) according to manufactures instructions. Spectrophotometrical absorbance of the samples were measured and the total absorbance was calculated by subtraction of delta blanked values (475 nm) with their reference values at 650 nm (E, n=5), (F, n=3). (**G**, **H**) The amounts of apoptotic cells were determined by staining with Annexin

V and propidium iodide and subsequently analyzing by flow cytometry on a FACScantoII (BD Biosciences) (G, n=3), (H, n=4).

The data presented are derived from 3 (**A**, **B**, **C**, **D**, **F**, **G**), 5 (**E**) or 4 (**H**) independent experiments, each comprising 3 technical replicates for all indicated samples.

Ordinary one-way ANOVA followed by Dunnett's test to correct for multiple comparison.

* p<0.05, ** p<0.01, *** p<0.001, **** p<0.0001, ns indicates no significance.

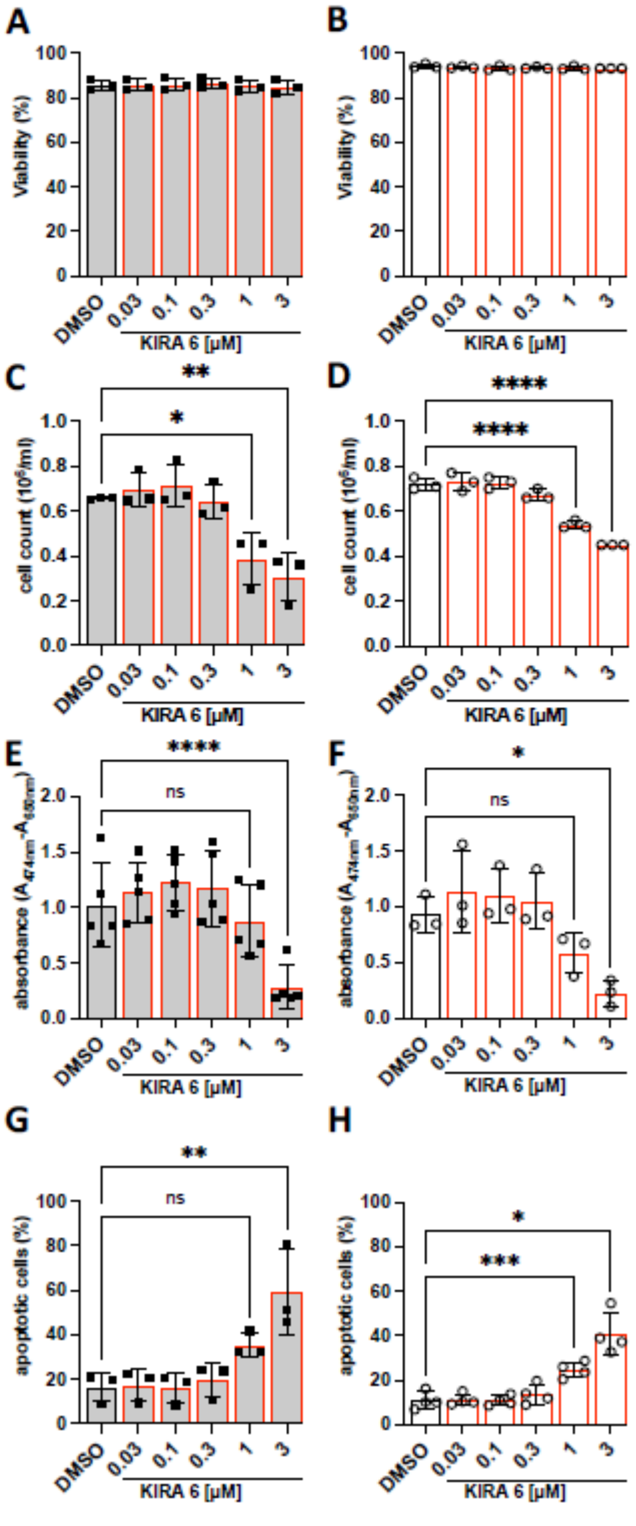
A
B
C
D
E
F
G
H
Viability (%)
cell count (10^6/ml)
absorbance (A_{474nm}-A_{650nm})
apoptotic cells (%)
DMSO
0.03
0.1
0.3
1
3
KIRA 6 [µM]
*
**
***
****
ns
